# Tuberculosis and antimicrobial resistance – new models of research and development needed

**DOI:** 10.2471/BLT.17.194837

**Published:** 2017-05-01

**Authors:** Grania Brigden, José Luis Castro, Lucica Ditiu, Glenda Gray, Debra Hanna, Marcus Low, Malebona Precious Matsoso, Greg Perry, Melvin Spigelman, Souyma Swaminathan, Els Torreele, Sidney Wong

**Affiliations:** a The International Union Against TB and Lung Disease, 68 Boulevard Saint-Michel, 75006, Paris, France.; b Stop TB Partnership, Geneva, Switzerland.; c South African Medical Research Council, Cape Town, South Africa.; d Critical Path Institute, Tucson, United States of America (USA).; e Global Tuberculosis Community Advisory Board, Cape Town, South Africa.; f Department of Health, Pretoria, South Africa.; g Medicines Patent Pool, Geneva, Switzerland.; h Global Alliance for TB Drug Development, New York, USA.; i Indian Council of Medical Research, New Delhi, India.; j Médecins Sans Frontières Access Campaign, Geneva, Switzerland.; k Médecins Sans Frontières, Amsterdam, Netherlands.

Tuberculosis is a disease that needs more investment in research and development. More people – 1.4 million in 2015 – die from tuberculosis every year than from human immunodeficiency virus (1.1 million deaths; 400 000 die from combinations of these infections) and malaria (429 000 deaths). Despite a current global caseload of 580 000 people infected with drug-resistant tuberculosis,^1^ current levels of investment – 620 million United States dollars – in research and development are at their lowest since 2008.^2^ Over the past decade, only two new drugs have been licensed; bedaquiline and delamanid. Tuberculosis cannot be cured by a single drug, but requires at least three different classes of antibiotic for treatment. Drug-resistant tuberculosis – bacilli resistant to two or more of the available antibiotics – is a persistent problem and is projected to account for 25% of deaths from all drug-resistant pathogens in the future.^3^

In 2010, the World Health Organization’s (WHO’s) Consultative Expert Working Group on Research and Development was established to examine current financing and incentives for research and development and to propose new approaches addressing unmet medical needs. Delegates at this month’s World Health Assembly will continue discussions to implement the recommendations from the group’s 2012 report on global financing and coordination of research and development.^4^

A United Nations (UN) General Assembly session on antimicrobial resistance and the UN High Level panel on Access to Medicines,^5^ as well as reports from the United Kingdom of Great Britain and Northern Ireland^3^ and the German government^6^ have all looked at new research and development models to incentivize research for drug-resistant infections.

Several nongovernmental organizations, medical research councils, civil society representatives and the South African government have recently developed a new funding framework to support research and development of tuberculosis treatments – the 3P Project (pull, pool and push). This initiative (i) uses a *pull* incentive, by rewarding research through prizes; (ii) *pools* intellectual property and data; and (iii) uses *push* incentives through research grants.^7^ The 3P project is a collaborative research initiative that aims to support the discovery and development of a one-month treatment regimen that can be used to cure all cases of tuberculosis. The project’s funding model will ensure that a new regimen is affordable and accessible to all those in need. The 3P Project incentivizes researchers by providing cash prizes for compounds that meet predefined product characteristics and are ready to enter phase I clinical trials. Coupling this financial reward with an obligation to pool the compounds data and intellectual property, the 3P Project will then fund the development of treatment combinations. The project will thus de-link the costs of research and development from the final cost of the treatment and sales as defined by the UN *Political declaration of the high-level meeting of the General Assembly on antimicrobial resistance*;^8^ ensuring treatment affordability.

We suggest that new models of research and development for tuberculosis, like the 3P Project, should be included in inter-governmental discussions on antimicrobial resistance priority setting. We encourage member states to support this initiative as a proactive response to address the priority pathogen for antimicrobial resistance.
